# Exposure to domestic violence and abuse and consultations for emergency contraception: nested case-control study in a UK primary care dataset

**DOI:** 10.3399/bjgp18X700277

**Published:** 2018-12-04

**Authors:** Joni Jackson, Natalia V Lewis, Gene S Feder, Penny Whiting, Timothy Jones, John Macleod, Maria Theresa Redaniel

**Affiliations:** National Institute for Health Research Collaboration for Leadership in Applied Health Research and Care West (NIHR CLAHRC West), University Hospitals Bristol NHS Foundation Trust, and Population Health Sciences, Bristol Medical School, University of Bristol, Bristol.; Professor in clinical epidemiology and primary care Centre for Academic Primary Care, Population Health Sciences, Bristol Medical School, University of Bristol, Bristol.; Professor in clinical epidemiology and primary care Centre for Academic Primary Care, Population Health Sciences, Bristol Medical School, University of Bristol, Bristol.; National Institute for Health Research Collaboration for Leadership in Applied Health Research and Care West (NIHR CLAHRC West), University Hospitals Bristol NHS Foundation Trust, and Population Health Sciences, Bristol Medical School, University of Bristol, Bristol.; National Institute for Health Research Collaboration for Leadership in Applied Health Research and Care West (NIHR CLAHRC West), University Hospitals Bristol NHS Foundation Trust, and Population Health Sciences, Bristol Medical School, University of Bristol, Bristol.; Centre for Academic Primary Care, Population Health Sciences, Bristol Medical School, University of Bristol, Bristol.; National Institute for Health Research Collaboration for Leadership in Applied Health Research and Care West (NIHR CLAHRC West), University Hospitals Bristol NHS Foundation Trust, and Population Health Sciences, Bristol Medical School, University of Bristol, Bristol.

**Keywords:** contraception, domestic violence, emergency contraception, general practice, intimate partner violence, postcoital, primary health care

## Abstract

**Background:**

Evidence of an association between exposure to domestic violence and abuse (DVA) and use of emergency contraception (EC) is lacking in the UK.

**Aim:**

To quantify the association between exposure to DVA and consultations for EC in general practice.

**Design and setting:**

Nested case-control study in UK general practice.

**Method:**

Using the Clinical Practice Research Datalink, the authors identified all women all women aged 15–49 years registered with a GP between 1 January 2011 and 31 December 2016. Cases with consultations for EC (*n* = 43 570) were each matched on age and GP against four controls with no consultations for EC (*n* = 174 280). The authors calculated odds ratios (ORs) and 95% confidence intervals (CIs) for the association between exposure to DVA in the previous year and consultations for EC. Covariates included age, ethnicity, socioeconomic status, pregnancy, children, alcohol misuse, and depression.

**Results:**

Women exposed to DVA were 2.06 times more likely to have a consultation for EC than unexposed women (95% CI = 1.64 to 2.61). Women aged 25–39 years with exposure to DVA were 2.8 times more likely to have a consultation for EC, compared with unexposed women (95% CI = 2.08 to 3.75). The authors found some evidence of an independent effect of exposure to DVA on the number of consultations for EC (OR 1.48, 95% CI = 0.99 to 2.21).

**Conclusion:**

A request for EC in general practice can indicate possible exposure to DVA. Primary care consultation for EC is a relevant context for identifying and responding to DVA as recommended by the World Health Organization and National Institute for Health and Care Excellence guidelines. DVA training for providers of EC should include this new evidence.

## INTRODUCTION

Domestic violence and abuse (DVA) encompass *‘any incident or pattern of incidents of controlling, coercive, threatening behaviour, violence or abuse between those aged 16 or over who are, or have been, intimate partners or family members’*.^1^ Although experienced across the sexes, health consequences of DVA are reported as being worse among women, mainly impacting on their mental and reproductive health.^2^ Two systematic reviews have found an association between exposure to DVA and reduction in use of regular contraception.^3^^,^^4^ The authors’ recent systematic review found some evidence for a positive association between exposure to DVA and use of emergency contraception (EC).^5^ However, none of these reviews included UK studies.

The negative impact of DVA on health results in higher presentation of women exposed to DVA among healthcare service users compared with general populations. Between 7 and 17% of female patients in general practice reported experiencing DVA in the previous year.^6^^–^8 National and international health organisations identify primary care providers as an important point of contact for victims of DVA and survivors.^9^^–^12 Patients perceive healthcare professionals as being well placed to enquire about DVA and respond to disclosure.^13^ World Health Organization (WHO)^12^ and National Institute for Health and Care Excellence (NICE)^11^ guidelines recommend a case-finding or clinical enquiry approach to DVA identification, prompted by clinical conditions associated with DVA. Recommended initial response to disclosure should follow the WHO LIVES principles: Listen, Inquire about needs and concerns, Validate, Enhance safety, provide Support.^14^ A number of DVA resources for healthcare professionals are available in the UK,^15^^–^18 including the Identification and Referral to Improve Safety (IRIS) model — a training, support, and referral programme for general practice^19^^–^22 and sexual health services,^23^^,^^24^ currently implemented across 30 administrative areas. None of the existing DVA training sources includes presentation for EC as a condition associated with DVA that should trigger clinical enquiry. As the association between DVA and use of EC can be influenced by the country context, such as access to EC,^25^^,^^26^ it is important to obtain the UK evidence to inform national clinical guidance and training resources on DVA. New evidence will inform clinicians’ decision making on DVA clinical enquiry, and potentially lead to more women with experience of DVA accessing evidence-based interventions.

**How this fits in**

Professional awareness of clinical associations of domestic violence and abuse (DVA) is a first step towards the evidence-based healthcare response recommended by the World Health Organization and National Institute for Health and Care Excellence. This study’s findings fill the gap in evidence from UK primary care on the association between exposure to DVA and increased use of emergency contraception (EC). This study found that a request for EC in general practice can indicate possible exposure to DVA. A consultation for EC is an appropriate context for asking about DVA, responding supportively, and offering referral to specialist DVA services.

This study aimed to fill a gap in the UK-based evidence by quantifying the association between exposure to DVA and general practice consultation for EC. The primary objective was to estimate the association between exposure to DVA and consultations for EC. The secondary objective was to estimate whether there is an association between exposure to DVA and having multiple consultations for EC. The authors hypothesised that exposure to DVA is associated with an increase in EC consultations.

## METHOD

### Study design

The authors conducted a nested case-control study in the Clinical Practice Research Datalink (CPRD), which contains anonymised electronic primary care records for approximately 17 million patients registered at 718 participating general practices in the UK.^27^ Patients registered during any time period and meeting quality criteria monitored by the CPRD (about 15 million) are considered broadly representative of the UK population with regards to age, sex, and ethnicity. The time from the date when practice data were considered to meet CPRD standards for quality and completeness is defined as the period of up-to-standard (UTS) registration. CPRD data are recorded by general practice clinicians using version 2 Read codes, a hierarchical clinical classification system containing >96 000 codes.^28^ Prescriptions are automatically recorded with a product name and *British National Formulary* code.

The authors used CPRD data on patient demographics, consultations (medical codes), and prescriptions (product codes) recorded by clinicians as part of their usual medical practice. For each variable, one researcher ran searches and compiled a draft list of Read and drug codes,^29^ which was revised by two academic GPs and cross-referenced with comparable code lists from the online clinical codes repository^30^ and the authors’ previous studies,^19^ leading to the final version of codes for data extraction (further details are available from the authors on request).

### Participants

The authors identified female patients aged 15–49 years (the WHO definition of reproductive age)^31^ with any period of registration at a general practice between 1 January 2011 and 31 December 2016. From this cohort, the authors identified all women with at least one record of EC consultation within the study period (cases). The date of the first EC consultation was defined as the case’s index date (or index consultation).

Each case was matched on age (year of birth ± 2 years) and general practice, with up to four controls who had no record of EC consultation within the study period and were randomly selected from the study population. Controls inherited the index date of their matched case. All patients were required to have at least 1 complete year of UTS medical history before their index date.

Potential controls were excluded if they had any indication that they would not have been eligible for, or needed, EC. The full list of inclusion and exclusion criteria, with justification, is available from the authors on request.

### Variables

#### Exposure and outcomes

Primary outcome (an index consultation for EC) was the first occasion on which any EC Read or drug code from the authors’ list was entered in the patient medical record. Secondary outcome (multiple consultation for EC) was defined as having more than one consultation for EC, occurring >1 week apart and within 12 months after the index consultation (to increase the likelihood that records relate to separate events occurring within a relatively short time period).

Exposure to DVA was defined as the first retrospective record of any CPRD code for DVA within 12 months before the index consultation for EC, to meet the UK Home Office definition of DVA and allow an adequate window for exposure to affect the outcome (further details are available from the authors on request.

#### Covariates

The authors extracted CPRD and linkage data on known factors that affect women’s use of contraception.^4^ CPRD covariates included age (year of birth) and history of alcohol abuse (Read codes for any records of alcoholism, alcohol dependency, or alcohol induced disease since the age of 15 years), or depression (Read and drug codes for any records of depression or depressive episode within 2 years before the index date)^30^ (further details are available from the authors on request).

Other covariates were extracted from datasets linked to CPRD. Information about patient ethnicity (white, black, Asian, mixed, other) was obtained via linkage to the Hospital Episode Statistics (HES) dataset. Socioeconomic status (quintiles of Indices of Multiple Deprivation [IMD]) was identified via linkage to the 2015 English IMD^32^ (2011 lower layer super output area [LSOA] boundaries). Information about number of pregnancies was obtained from the CPRD pregnancy register, which contains details about all pregnancies identified in the CPRD using an algorithm developed to identify and maximise the use of records relating to the timing and duration of pregnancy, the type of pregnancy outcome (live birth, stillbirth, or pregnancy loss), and additional features pertaining to the pregnancy. The authors identified the number of children for each woman using the CPRD mother–baby link, which contains data on mother–baby pairings, linked using an algorithm that matches live births to maternal records in the CPRD. No restrictions relating to use of data from UTS time only within a practice are imposed, and therefore may include children born before the practice became UTS and those who initially registered at a different practice after birth, but subsequently joined the current practice.

### Statistical analysis

Statistical analyses were performed using STATA version 15. The authors used univariable and multivariable conditional logistic regression models to estimate odds ratios (ORs) and 95% confidence intervals (CIs) for the association between record for DVA exposure and consultations for EC. The choice of analysis allowed the authors to take into account matched sets of cases and controls, among whom unmeasured confounders are assumed equal.

Multiple imputation for missing ethnicity or socioeconomic status was not performed, as the necessary data to inform the imputation were not available. The authors conducted an analysis of all subjects, excluding ethnicity and socioeconomic status from the model, and an analysis of complete cases adjusting for all covariates.

Secondary analysis used univariable and multivariable logistic regression to model the association between exposure to DVA and multiple consultations for EC among cases.

## RESULTS

**Figure 1.**
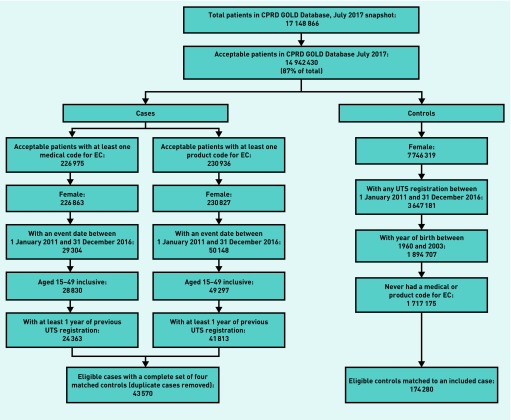
***Flow diagram reporting numbers of patients at each stage of the study.*** ***CPRD = Clinical Practice Research Datalink.*** ***EC = emergency contraception. UTS = up to standard.***

The authors identified 43 570 eligible cases and 174 280 matched controls (Figure 1).

Cases and controls were similar in terms of age, ethnicity, and socioeconomic status (Table 1). Cases tended to have had more pregnancies and to have more children, and were more likely to have a history of depressive episodes.

**Table 1.** Demographic and patient characteristics of women who had a consultation for emergency contraception (cases), and women who did not have a consultation for emergency contraception (controls)

**Variable**		**Cases, *n* (%)**	**Controls, *n* (%)**
Previous 12 months’ experience of domestic violence and abuse^a^	No	43 418 (99.65)	174 108 (99.90)
	Yes	152 (0.35)	172 (0.10)

Mean age, years (standard deviation)		28.46 (8.29)	28.46 (8.41)

Age categories, years	15–24	15 844 (36.36)	63 510 (36.44)
	25–39	22 545 (51.74)	89 410 (51.30)
	40–49	5181 (11.89)	21 360 (12.26)

Ethnicity	White	17 986 (41.28)	56 450 (32.39)
	Black	1001 (2.30)	2033 (1.17)
	Asian	1065 (2.44)	3848 (2.21)
	Mixed	389 (0.89)	800 (0.46)
	Other	354 (0.81)	1370 (0.79)
	Missing	22 775 (52.27)	109 779 (62.99)

Socioeconomic status (quintiles of level of deprivation)	Least deprived: 1	4419 (10.14)	19 991 (11.47)
	2	4665 (10.71)	19 795 (11.36)
	3	5140 (11.80)	20 252 (11.62)
	4	5437 (12.48)	19 813 (11.37)
	Most deprived: 5	5615 (12.89)	18 959 (10.88)
	missing	18 294 (41.99)	75 470 (43.30)

Pregnancies, *n*	0	15 617 (35.84)	105 190 (60.36)
	1	7209 (16.55)	24 178 (13.87)
	≥2	20 744 (47.61)	44 912 (25.77)

Children, *n*	0	25 426 (58.36)	133 040 (76.34)
	1	10 623 (24.38)	25 472 (14.62)
	≥2	7521 (17.26)	15 768 (9.05)

History of alcohol misuse since age 15^a^	No	43 180 (99.10)	173 621 (99.62)
	Yes	390 (0.90)	659 (0.38)

History of depression/depressive episode within 2 years^a^	No	39 174 (89.91)	166 101 (95.31)
	Yes	4396 (10.09)	8179 (4.69)

a Before the index date.

In the year before the index date, 0.35% of cases and 0.10% of controls had one or more record of DVA (Table 1). Compared with women with no record of DVA, women who had experienced DVA within the year before their index date were more than three times likely to have had one or more consultations for EC (OR 3.59, 95% CI = 2.88 to 4.47) (Table 2).

**Table 2.** Association of domestic violence and abuse and other covariates with having a consultation for emergency contraception, univariable analysis

**Variable**		**All subjects (*n* = 217 850)**		**Cases with complete data (*n* = 22 135)**	
	
		**OR (95% CI)**	***P*-value**	**OR (95% CI)**	***P*-value**
Previous 12 months’ experience of domestic violence and abuse^a^	No	1.00		1.00	
	Yes	3.59 (2.88 to 4.47)	<0.000	3.37 (1.95 to 5.81)	<0.000

Age categories, years	15–24	0.98 (0.92 to 1.04)	0.415	0.93 (0.75 to 1.16)	0.527
	25–39	1.00		1.00	
	40–49	0.78 (0.72 to 0.85)	<0.000	0.84 (0.69 to 1.04)	0.109

Ethnicity	White	1.00		1.00	
	Black	1.60 (1.46 to 1.73)	<0.000	1.42 (1.17 to 1.71)	<0.000
	Asian	0.87 (0.81 to 0.94)	<0.000	0.87 (0.74 to 1.03)	0.099
	Mixed	1.51 (1.33 to 1.71)	<0.000	1.42 (1.07 to 1.90)	0.017
	Other	0.81 (0.71 to 0.91)	<0.000	0.61 (0.45 to 0.82)	0.001
	Missing	0.37 (0.36 to 0.38)	<0.000		

Socioeconomic status (quintiles of level of deprivation)	Least deprived: 1	1.00		1.00	
	2	1.14 (1.09 to 1.20)	<0.000	1.07 (0.94 to 1.21)	0.314
	3	1.30 (1.23 to 1.36)	<0.000	1.29 (1.14 to 1.47)	<0.000
	4	1.49 (1.41 to 1.57)	<0.000	1.36 (1.20 to 1.55)	<0.000
	Most deprived: 5	1.74 (1.64 to 1.84)	<0.000	1.66 (1.45 to 1.91)	<0.000
	Unknown	0.36 (0.32 to 0.41)	<0.000		

Pregnancies, *n*	0	1.00		1.00	
	1	2.93 (2.83 to 3.04)	<0.000	2.23 (1.98 to 2.52)	<0.000
	≥2	5.56 (5.39 to 5.74)	<0.000	4.39 (3.95 to 4.88)	<0.000

Children, *n*	0	1.00		1.00	
	1	2.63 (2.55 to 2.71)	<0.000	1.82 (1.68 to 1.97)	<0.000
	≥2	3.27 (3.15 to 3.38)	<0.000	2.18 (1.99 to 2.39)	<0.000

History of alcohol misuse since age 15^a^	No	1.00		1.00	
	Yes	2.40 (2.11 to 2.72)	<0.000	2.58 (1.83 to 3.64)	<0.000

History of depression/depressive episode within 2 years^a^	No	1.00		1.00	
	Yes	2.32 (2.24 to 2.42)	<0.000	1.98 (1.77 to 2.22)	<0.000

a Before index date.

When included individually, potential confounding factors changed odds ratios for exposure to DVA by percentages ranging from 0.8% to 38.2%, with number of pregnancies producing the largest change. After adjusting for all covariates, women who had been exposed to DVA were two times more likely to have a consultation for EC than women with no experience of DVA (OR 2.06, 95% CI = 1.64 to 2.61) (Table 3).

**Table 3.** Association of domestic violence and abuse with having a consultation for emergency contraception, multivariable conditional logistic regressions models

**Model**		**All subjects (*n* = 217 850)**		**Cases with complete data (*n* = 22 135)**	
	
		**Adjusted OR (95% CI)**	***P*-value**	**Adjusted OR (95% CI)**	***P*-value**
Previous 12 months DVA^a^		3.59 (2.88 to 4.47)	<0.000	3.37 (1.95 to 5.81)	<0.000

Previous 12 months DVA^a^ and age categories		3.59 (2.88 to 4.47)	<0.000	3.38 (1.96 to 5.84)	<0.000

Previous 12 months DVA^a^ and ethnicity				3.43 (1.99 to 5.94)	<0.000

Previous 12 months DVA^a^ and socioeconomic status				3.29 (1.90 to 5.70)	<0.000

Previous 12 months DVA^a^ and number of pregnancies		2.22 (1.76 to 2.80)	<0.000	2.76 (1.57 to 4.85)	<0.000

Previous 12 months DVA^a^ and number of children		2.85 (2.27 to 3.58)	<0.000	3.23 (1.85 to 5.61)	<0.000

Previous 12 months DVA^a^ and record of alcohol abuse^b^		3.54 (2.84 to 4.41)	<0.000	3.20 (1.84 to 5.54)	<0.000

Previous 12 months DVA^a^ and record of depression/depressive episode^c^		3.28 (2.63 to 4.10)	<0.000	3.12 (1.80 to 5.42)	<0.000

Full model		2.06 (1.64 to 2.61)	<0.000	2.53 (1.43 to 4.47)	0.001

Full model and previous 12 months DVA^a^ multiplied by age interaction	15–24 years	1.25 (0.82 to 1.93)	0.290		
	25–39 years	2.78 (2.08 to 3.75)	<0.000		
	40–49 years	1.23 (0.51 to 3.02)	0.643		

a Before index date.

b Since age 15, before index date.

c Within 2 years before index date. DVA = domestic violence and abuse. OR = odds ratio.

There was a positive interaction between exposure to DVA and age, with the odds of EC consultation being 2.8 times greater among women aged 25–39 years with exposure to DVA, compared with those without such exposure (OR 2.78, 95% CI = 2.08 to 3.75) (Table 3).

When restricted to women with complete data for all covariates, the overall pattern of results was similar to the main analysis, that is, women who had been exposed to DVA were more than twice as likely to have a consultation for EC than women with no experience of DVA (Tables 2 and 3).

In the 12 months following their index date, 12.8% of cases had multiple consultations for EC. Demographic and patient characteristics were similar between cases who had a single consultation and cases who had multiple consultations for EC (Table 4).

**Table 4.** Demographic and patient characteristics of cases who had multiple consultations for emergency contraception and women who had a single consultation for emergency contraception

**Variable**		**Cases with a single consultation, *n* (%)**	**Cases with multiple consultations, *n* (%)**
Previous 12 months domestic violence and abuse^a^	No	37 869 (99.68)	5549 (99.46)
	Yes	122 (0.32)	30 (0.54)

Age categories, years	15–24	13 693 (36.04)	2151 (38.56)
	25–39	19 616 (51.63)	2929 (52.50)
	40–49	4682 (12.32)	499 (8.94)

Pregnancies, *n*	0	13 927 (36.66)	1690 (30.29)
	1	6204 (16.33)	1005 (18.01)
	≥2	17 860 (47.01)	2884 (51.69)

Children, *n*	0	22 266 (58.61)	3160 (56.64)
	1	9140 (24.06)	1483 (26.58)
	≥2	6585 (17.33)	936 (16.78)

History of alcohol misuse since age 15^a^	No	37 822 (99.56)	5550 (99.48)
	Yes	169 (0.44)	29 (0.52)

History of depression/depressive episode within 2 years^a^	No	34 261 (90.18)	4913 (88.06)
	Yes	3730 (9.82)	666 (11.94)

a Before the index date.

In the 12 months before their index date, 0.32% of cases with a single consultation for EC had one or more record of DVA, compared with 0.54% of cases with multiple consultations for EC (Table 4). Compared with women with no record of DVA, women who had experienced DVA within the 12 months before their index date were 1.7 times more likely to have had multiple consultations for EC (OR 1.68, 95% CI = 1.12 to 2.50) (Table 5).

**Table 5.** Association of domestic violence and abuse and other covariates with having multiple consultations for emergency contraception, univariable analysis

**Variable**		**OR (95% CI)**	***P*-value**
Previous 12 months domestic violence and abuse^a^	No	1.00	
	Yes	1.68 (1.12 to 2.50)	0.011

Age categories, years	15–24	1.05 (0.99 to 1.12)	0.096
	25–39	1.00	
	40–49	0.71 (0.65 to 0.79)	0.000

Pregnancies, *n*	0	1.00	
	1	1.33 (1.23 to 1.45)	0.000
	≥2	1.33 (1.25 to 1.42)	0.000

Children, *n*	0	1.00	
	1	1.14 (1.07 to 1.22)	0.000
	≥2	1.00 (0.93 to 1.08)	0.969

History of alcohol abuse within 2 years^a^	No	1.00	
	Yes	1.17 (0.79 to 1.74)	0.437

History of depression/depressive episode within 2 years^a^	No	1.00	
	Yes	1.25 (1.14 to 1.36)	0.000

a Before the index date. OR = odds ratio.

The addition of potential confounding factors to the model individually produced little change in odds ratios for exposure to DVA. When adjusted for all covariates, there was some evidence of an independent effect of exposure to DVA on the number of consultations for EC (OR 1.48, 95% CI = 0.99 to 2.21) (Table 6).

**Table 6.** Association of domestic violence and abuse with having multiple consultations for emergency contraception, multivariable logistic regressions models

**Model**		**Adjusted OR (95% CI)**	***P*-value**
Previous 12 months DVA^a^		1.68 (1.12 to 2.50)	0.011
Previous 12 months DVA^a^ and age categories		1.65 (1.11 to 2.46)	0.014
Previous 12 months DVA^a^ and number of pregnancies		1.57 (1.05 to 2.34)	0.028
Previous 12 months DVA^a^ and number of children		1.66 (1.11 to 2.48)	0.013
Previous 12 months DVA^a^ and record of alcohol abuse^b^		1.67 (1.12 to 2.50)	0.012
Previous 12 months DVA^a^ and record of		1.65 (1.11 to 2.47)	0.014
depression/depressive episode^c^			
Full model		1.48 (0.99 to 2.21)	0.058
Full model and previous 12 months DVA^a^ multiplied by age interaction	15–24	0.96 (0.43 to 2.17)	0.923
	25–39	1.67 (1.03 to 2.70)	0.038
	40–49	3.44 (0.67 to 17.81)	0.140

a Before index date.

b Since age 15, before index date.

c Within 2 years before index date. DVA = domestic violence and abuse. OR = odds ratio.

The positive interaction between exposure to DVA and age remained, with the odds of having multiple consultations for EC being 1.7 times greater among women aged 25–39 years with exposure to DVA, compared to those without such exposure (OR 1.67, 95% CI = 1.03 to 2.70) (Table 6).

## DISCUSSION

### Summary

In this nested case-control study of UK general practice data, after adjusting for covariates, women with exposure to DVA within the past 12 months were two times more likely to have had at least one consultation for EC, compared with women with no exposure to DVA. There was also some evidence that, after adjusting for covariates, women with exposure to DVA within the past 12 months were >1.5 times more likely to have had multiple consultations for EC, compared with women without such exposure.

### Strengths and limitations

The main strengths of this study are the coverage and quality of the CPRD data, and the representativeness of included cases. CPRD data are entered by clinicians during routine consultations in general practice, rather than for research purposes. As practice-based prescribing is generally electronic, prescriptions for EC will automatically be captured by GP software systems, leading to good ascertainment of cases. In addition, data quality for patients and practices are monitored by CPRD internal processes for validity and completeness, and active patients (as are included in this study) are generally representative of the UK population in terms of age and sex.^27^

This study’s main limitation is the under-recording of DVA in electronic medical records, which could attenuate the association between exposure to DVA and consultations for EC. The first UK study on DVA in primary care found an 83% under-recording of DVA exposure when comparing rates in electronic medical records with patient’s self-reported rates.^33^ Another study found that, although multiple Read codes exist for DVA, recording practices vary considerably across UK general practice.^34^ Additionally, there is a potential misclassification of controls, due to the increasing availability of EC from varied providers during the past 10 years.^35^ Though the authors’ cases sought EC through general practice, the controls could either have not used EC (true controls) or obtained it elsewhere (misclassified controls). The authors also did not adjust their models for misuse of substances other than alcohol, known to be associated with DVA.^36^ Their findings are also restricted to consultations for EC provided by clinicians in general practice. Data from other sources of EC provision, such as sexual and reproductive health services and community pharmacies, could not be linked to CPRD due to regulatory, technical, and logistical reasons. The authors estimate that they have captured around 30% of the total provision of EC.^37^ Considering these limitations, the authors anticipate that the true association between DVA and EC is higher than the results they have presented here.

### Comparison with existing literature

This study supports the recent systematic review of cross-sectional studies that found some evidence for an association between exposure to DVA and increased use of EC.^5^ The two main reasons for seeking EC are women’s fear that the contraceptive method they used would not work, and women’s fear of unintended pregnancy after unprotected intercourse.^38^ A recent meta-analysis suggested a causal relationship between exposure to DVA and reduction in women’s use of regular contraception.^4^ The authors can further speculate that, due to the reduction in use of regular contraception, women exposed to DVA are more likely to have unprotected intercourse and therefore might need more EC compared with women unexposed to DVA. Another mechanism connecting DVA and unprotected intercourse is reproductive coercion — a pattern of male behaviour aimed at controlling women’s reproductive outcomes through birth control sabotage or pregnancy coercion.^39^^,^^40^

The authors’ findings are in line with the US study on the effect of DVA on contraceptive patterns, though their effect estimates are much smaller.^41^ Fantasia *et al* analysed medical records from four family planning clinics and found that exposure to DVA in the previous 12 months was associated with a 6.5-fold increase in use of EC (95% CI = 3.8 to 9.3).^41^ Several factors could contribute to the difference in the effect size: study settings, national differences in the provision of EC, and methods of identifying and recording exposure, outcome, and confounding variables.

The authors found some evidence that women exposed to DVA can seek EC from GPs on multiple occasions. This suggests women exposed to DVA may use EC instead of regular contraceptive methods, which they cannot access because of reproductive coercion, coercive control, or economic abuse. This is in line with the US survey in five family planning clinics which showed that women who had experienced DVA and reproductive coercion were two times more likely to report multiple use of EC in the previous 3 months (OR 2.40, 95% CI = 1.41 to 4.09).^42^ Reproductive coercion was not captured in the present study, which could have resulted in the smaller effect estimate. However, the authors considered the temporality of the exposure versus outcome, which was not possible in the US study.^42^

### Implications for research and practice

Future studies should use data linkage from core providers of EC (general practice, sexual and reproductive health services, and pharmacies) to analyse the total provision of EC.

DVA interventions for primary care (for example, IRIS in the UK) should be updated to include new evidence on the association between exposure to DVA and increased use of EC. All providers of EC should be aware that a request for EC can indicate possible exposure to DVA. A consultation for EC in general practice is an appropriate context for asking about DVA and responding to disclosure in line with the WHO LIVES^14^ principles.
